# In Vitro Larvicidal Efficacy of a Fipronil-Based Nanoixodicide Against *Rhipicephalus microplus*

**DOI:** 10.3390/tropicalmed10100284

**Published:** 2025-10-06

**Authors:** José Pablo Villarreal-Villarreal, José Noel García-Pérez, Jesús Jaime Hernández Escareño, Sergio Arturo Galindo Rodríguez, Michel Stéphane Heya, Gustavo Hernández Vidal, Romario García-Ponce

**Affiliations:** 1 Facultad de Medicina Veterinaria y Zootecnia, Universidad Autónoma de Nuevo León, General Escobedo 66054, México; pablo.villarrealvl@uanl.edu.mx (J.P.V.-V.); jose.garciapre@uanl.edu.mx (J.N.G.-P.); jesus.hernandezec@uanl.edu.mx (J.J.H.E.); 2 Facultad de Ciencias Biológicas, Universidad Autónoma de Nuevo León, San Nicolás de los Garza 66455, Mexico; sergio.galindord@uanl.edu.mx; 3 Facultad de Salud Publica y Nutrición, Universidad Autónoma de Nuevo León, Monterrey 64460, Mexico; michel.heyax@uanl.edu.mx

**Keywords:** polymeric nanoparticles, nanoixodicide, fipronil, *Rhipicephalus microplus*

## Abstract

Controlling *Rhipicephalus microplus* is currently one of the main challenges in livestock farming due to the significant economic losses it causes. Traditionally, managing this parasite has been based on the use of synthetic ixodicides, among which fipronil has proven to be highly effective. However, its low water solubility and the limitations of commercially available formulations can affect the bioavailability of this compound, favoring the emergence of resistance in tick populations. In this context, fipronil-loaded nanoparticles were developed using the Eudragit^®^ E PO polymer (NP_F) (Helm, Naucalpan, Mexico, Mexico), which were physicochemically characterized and evaluated against fipronil-susceptible *R. microplus* larvae. NP_F had an average size of 143.43 ± 1.88 nm, a polydispersity index (PDI) of 0.162 ± 0.01, a ζ (P ζ) of 21.16 ± 0.54, an encapsulation percentage (%E) of 7.36 ± 0.30, and an encapsulation efficiency percentage (%EE) of 66.28 ± 3.5%. Free fipronil showed an LC_50_ of 0.582 µg/mL and an LC_90_ of 2.503 µg/mL against *R*. *microplus*. The NP_F formulation showed an LC_50_ of 0.427 µg/mL and an LC_90_ of 2.092 µg/mL. These results suggest that incorporating fipronil into nanoparticles improves its ixodicide efficacy, positioning it as an innovative and promising alternative for the development of effective tick control formulations.

## 1. Introduction

Worldwide, it is estimated that there are more than one billion cattle, of which approximately 90% are at risk of infestation by various species of ticks [1,2]. Among these, *Rhipicephalus microplus* stands out as the most significant and impactful species for livestock production due to its remarkable adaptability, which has allowed it to establish itself in various geographical regions throughout the world [3]. This blood-feeding species causes significant economic losses due to its direct effects, which include reduced body weight, anemia, skin lesions, decreased reproductive efficiency, and reduced milk production [4]. In addition, it acts as a vector for pathogens that cause diseases such as babesiosis and anaplasmosis [5,6]. In Mexico, *R*. *microplus* represents the main health problem for cattle, with annual economic losses exceeding $573.6 million [7].

The traditional control method involves synthetic ixodicides; however, their effectiveness is limited mainly by application errors, which have led to resistant tick populations [8,9,10]. Fipronil is an ixodicide belonging to the phenylpyrazole class, characterized by its potent action against ticks and other arthropods of importance in veterinary and agricultural industry. This compound was developed in the late 1980s and commercially launched in 1993; it represented a significant innovation in ectoparasite control due to its unique mechanism of action on GABA receptors and glutamate-regulated chloride channels in the nervous system of invertebrates. [11,12,13,14]. Since its introduction, fipronil has demonstrated high efficacy against a broad spectrum of arthropods, including ticks, fleas, mites, and various insect pests, which has favored its acceptance in control programs within both veterinary medicine and agriculture [11]. Nevertheless, it has technical limitations, such as its low water solubility, which hinders the development of safe and efficiently applicable formulations [15,16]. Currently, it is marketed in pour-on formulations; however, this method presents logistical challenges in large cattle herds, where product administration can be irregular, resulting in inadequate dosage. These factors contribute to the selection of resistant strains, which in turn forces an increase the dosing or the use of more expensive alternatives, thus complicating the management and control of infestations.

Furthermore, cases of resistance of *R. microplus* populations to fipronil have been documented in several countries, including Mexico [17], Uruguay [18,19], Brazil [20], Argentina [21], and India [22]. This resistance represents a significant threat to the efficacy of control programs, as it progressively reduces the usefulness of one of the most effective ixodicides available. Given this scenario, it is a priority for the livestock industry to develop and evaluate new formulation strategies that optimize the bioavailability and efficacy of fipronil, reducing administration failures, and helping to delay the development of resistance.

In this context, the use of polymeric nanoparticles as active ingredient carriers has gained great relevance in areas such as veterinary medicine and biotechnology [23,24,25,26]. Nanoparticles are nanometer-sized systems that have unique properties, such as their high surface area and the ability to overcome different biological barriers, allowing for their controlled release [27,28]. This makes them suitable for its use in drug delivery systems, thereby improving the physicochemical stability of encapsulated active ingredients and reducing the adverse effects of their conventional application [29,30,31,32]. Additionally, nanoparticles allow for the application of active ingredients in an aqueous medium, eliminating the need to use toxic organic solvents, which makes them a viable option for formulating and applying synthetic ixodicides [25].

Considering these advantages, the objective of this study focused on developing and characterizing a formulation of polymeric nanoparticles loaded with fipronil, evaluating its in vitro acaricidal activity against *R. microplus* larvae. This strategy seeks not only to improve the physicochemical stability and bioavailability of the compound but also to optimize its efficacy, reduce the necessary doses, and minimize the risks associated with conventional formulations.

## 2. Materials and Methods

### 2.1. Materials

Fipronil was acquired from Toronto Research Chemicals (Toronto, ON, Canada). The polymer Eudragit^®^ E PO was acquired from Helm (Naucalpan, Estado de Mexico, Mexico).

### 2.2. Preparation and Characterization of Polymeric Nanoparticles

**Figure 1:**
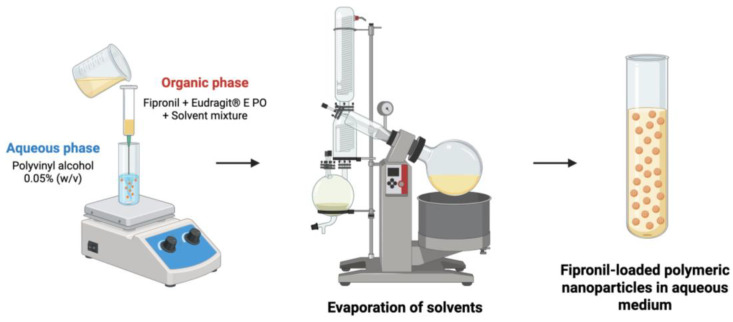
Obtaining of Eudragit^®^ E PO polymeric nanoparticles loaded with fipronil.

Fipronil-loaded nanoparticles were prepared using the nanoprecipitation technique [33]. Briefly, an organic phase composed of the polymer (Eudragit^®^ E PO) and fipronil dissolved in a solvent mixture (acetone, ethanol and isopropanol) was injected into an aqueous phase containing 0.05% *w*/*v* polyvinyl alcohol. The diffusion of the organic phase into the aqueous phase induced the polymer aggregation and the nanoparticle formation. Subsequently, the solvents were removed using a rotary evaporator (Laborota 4003 Control, Heidolph Instruments, Schawabach, Germany) and finally, the fipronil-loaded nanoparticles (NP_F) were obtained in aqueous solution (Figure 1). The nanoparticles without fipronil or white nanoparticles (NP_B) were obtained following the same procedure except for the active ingredient.

#### 2.2.1. Particle Size, Polydispersity Index (PDI) and ζ Potential (Pζ)

The particle size and polydispersity index (PDI) of the nanoparticle formulations were determined by dynamic light scattering at a 90° angle (Zetasizer Nano-ZS90, Malvern Instruments, Worcestershire, UK). The ζ-potential (Pζ) was evaluated by laser Doppler microelectrophoresis (Zetasizer Nano-ZS90, Malvern Instruments, Worcestershire, UK).

#### 2.2.2. Stability

The particle size, PDI and Pζ of the nanoformulations were monitored during 120 days of storage under ambient conditions, without protection from light.

#### 2.2.3. Fourier Transform Infrared (FT-IR) Analysis

The analysis of the Eudragit^®^ E PO polymer, fipronil, and the NP_B and NP_F formulations was performed using FT-IR spectroscopy. To identify potential molecular interactions, 64 scans were performed in the range of 4000 to 400 cm^−1^ with an FT-IR Optical Frontier spectrometer (PerkinElmer, Waltham, MA, USA).

#### 2.2.4. Quantification of Fipronil Incorporated into Nanoparticles

To determine the encapsulation efficiency percentage (%EE) and encapsulation percentage (%E) of fipronil in the formulation, a standard curve was prepared using fipronil solutions in a concentration range of 40 to 180 µg/mL.

To measure the fipronil incorporated into the nanoparticles, the aqueous dispersions were centrifuged at 33,600× *g* for 90 min, and detection was performed at 289 nm in a GENESYS 10S UV-Vis spectrophotometer (Thermo Scientific, Waltham, MA, USA). Each assay was performed in triplicate. Finally, the %EE and %E were determined using Formulas (1) and (2).

%EE = (mg of encapsulated fipronil/total mg of fipronil) ∗ 100(1)

%E = (mg of encapsulated fipronil/mg of polymer + total mg of fipronil) ∗ 100(2)

### 2.3. Collection and Establishment of the R. microplus Population

Engorged female *R. microplus* ticks were collected from naturally infested and previously untreated cattle from a production unit located in Tantoyuca, Veracruz, Mexico (21°21′07.6″ N, 98°14″ W). The specimens were manually removed, transported in plastic vials to the laboratory, and taxonomically identified based on the keys by Dantas-Torres et al. [34]. Subsequently, colonies were established at the Multidisciplinary Research Laboratory of the Faculty of Veterinary Medicine and Zootechnics at the Autonomous University of Nuevo Leon (Mexico). The ticks were washed, dried, and maintained at 28 ± 2 °C and 80–90% relative humidity to induce oviposition. The collected eggs were incubated under the same conditions until hatching, and larvae aged 7 to 14 days were used in the resistance diagnosis assays and bioassays.

### 2.4. Diagnosis of Fipronil Resistance

The resistance diagnosis of the tick population to fipronil was performed using the discriminating dose (DD) of fipronil at 0.05% *w*/*v* determined in Mexico by the National Center for Animal Health Services (CENA-PA-SENASICA-SADER) [35]. The Larval Immersion Test (LIT) was used for this purpose [36]. The assays were performed in triplicate, counting live and dead larvae [37]. The negative control consisted of 10% (*v*/*v*) methanol.

### 2.5. Bioassays with Fipronil-Loaded Nanoparticles

The ixodicidal activity of fipronil against *R. microplus* larvae was evaluated using the LIT [38], modified by Castro-Janer et al. [36]. For this, fipronil solutions were prepared at concentrations of 0.1, 0.2, 0.6, 1, 2, 3, and 5 μg/mL in 10% (*v*/*v*) methanol, and 1 mL of each concentration was transferred into 2 mL plastic tubes with caps. Approximately 500 larvae were immersed in each solution for 10 min, with manual agitation. Afterward, the tubes were drained, and the larvae were transferred to filter paper packets in groups of 100 to 200 individuals. The packets were incubated at 28 ± 2 °C and 80–90% relative humidity for 24 h, after which the number of live and dead larvae was recorded (Formula (3)).

The fipronil-loaded nanoparticles were evaluated using the same procedure, but at concentrations of 0.066, 0.132, 0.396, 0.66, 1.32, 1.98 and 3.3 μg/mL. As a negative control, nanoparticles such as fipronil (NP_B) were used. Larval mortality was corrected following FAO recommendations (2004) using Abbott’s formula [39] (Formula (3)). In cases where mortality in the control group exceeded 5%, the bioassay was invalidated and repeated. The obtained data were used to calculate the lethal concentrations at 50% and 90% (LC_50_ y LC_90_).

% Mortality = (% mortality in the test − % mortality in the control/100 − mortality in the control) ∗ 100(3)

### 2.6. Statistical Analysis

To estimate the LC_50_ y LC_90_, along with their 95% confidence intervals, a Probit analysis was performed using the Polo-Plus version 2.0 program (LeOra Software^®^). The analysis was based on the larval mortalities obtained at the different concentrations evaluated. It was considered that significant differences existed between the lethal concentrations when the 95% confidence intervals did not overlap.

## 3. Results

### 3.1. Characterization of the Nanoformulation

The NP_F formulation had a size of 143.43 ± 1.88 nm (Table 1). The nanoparticles without fipronil (NP_B) were smaller in size compared to the loaded nanoparticles. In both cases, the PDI was less than 0.2. Likewise, the Pζ of the nanoparticle formulation prepared with Eudragit^®^ E PO was 21.16 ± 0.54 mV (Table 1).

#### 3.1.1. Nanoforumulation Stability

**Figure 2:**
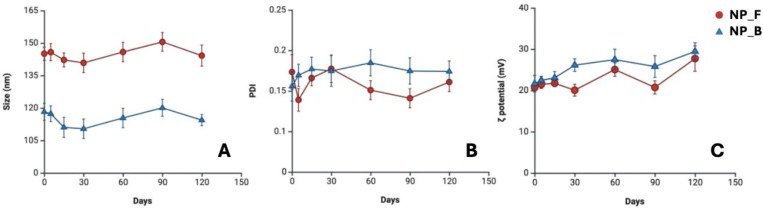
Stability of nanoparticle formulations over 120 days. (**A**) Particle size, (**B**) PDI, and (**C**) ζ potential of NP_F and NP_B. (mean ± SD, *n* = 3).

The size, PDI, and Pζ parameters of the NP_F and NP_B nanoformulations were evaluated over 120 days (Figure 2). During this period, both formulations remained stable, with no evidence of aggregation.

#### 3.1.2. FT-IR Analysis

**Figure 3:**
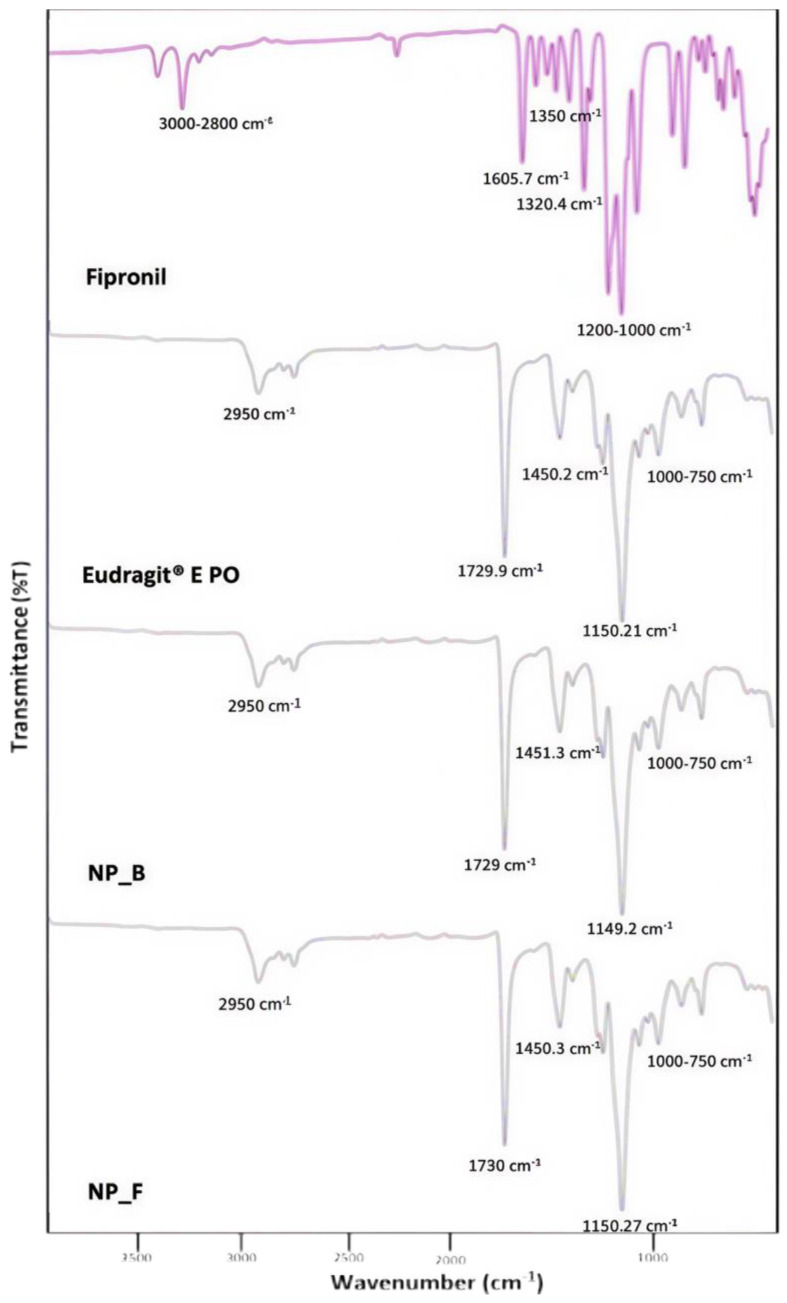
FT-IR spectra of fipronil, Eudragit^®^ E PO polymer, blank nanoformulation (NP_B), and fipronil-loaded nanoformulation (NP_F).

The spectra of the NP_F formulation were obtained and compared with those of fipronil, the Eudragit^®^ E PO polymer, and the blank nanoparticles (NP_B) to identify possible interactions or chemical reactions that occurred during the preparation process (Figure 3). In the fipronil spectrum, characteristic bands associated with its main functional groups were observed. In the 3000–2800 cm^−1^ region, weak signals corresponding to aliphatic C–H stretching were present. A strong band near 1605.7 cm^−1^ is attributed to the stretching of the aromatic ring in the compound’s structure. The characteristic vibrations of C–F bonds are evident in the 1200–1000 cm^−1^ region, where several strong, defined bands are located. Likewise, the sulfonyl group (–SO_2_–) shows intense absorptions around 1350–1150 cm^−1^, while vibrations associated with the C–N and C–O bonds appear between 1300–1000 cm^−1^. The Eudragit^®^ E PO polymer spectrum shows characteristic bands of its structure, which is based on acrylic and methacrylic derivatives. A signal at 2950 cm^−1^ is observed, attributed to the aliphatic C–H stretching of the methyl and methylene groups present in the polymeric chain. An intense band at 1730 cm^−1^ corresponds to the stretching of the carbonyl group (C=O), typical of acrylic and methacrylic esters. Additionally, the presence of a signal at 1150 cm^−1^ is associated with C–O–C stretching vibrations, confirming the existence of ester groups. The band recorded at 1450 cm^−1^ is related to the bending vibrations of C–H bonds. Finally, in the region between 1000 and 750 cm^−1^, more complex bands appear, corresponding to vibrations of the polymeric structure and deformations of C–C and C–H bonds. Lastly, the spectra of the NP_B and NP_F formulations showed a great similarity to the Eudragit^®^ E PO polymer spectrum (Figure 3).

### 3.2. Diagnosis of Fipronil Resistance

The resistance diagnosis showed that the *R. microplus* larval population exhibited 100% mortality when exposed to the DD established by CENAPA for fipronil. This result indicates that the population evaluated is susceptible to the compound, since it reached 100% mortality with the DD (Table 2).

### 3.3. Bioassays with Fipronil-Loaded Nanoparticles

As shown in Table 3, the ixodicidal activity of free fipronil against *R. microplus* larvae showed LC_50_ and LC_90_ values. Formulation NP_F achieved a lower LC_50_ than that of free fipronil. Formulation NP_B was used as a negative control and showed no ixodicidal activity.

## 4. Discussion

One of the main challenges associated with fipronil is its low water solubility (LogP = 3.75) [16], which limits its dispersion and bioavailability in aqueous media. This characteristic represents a significant obstacle to its formulation, as it hinders the development of presentations that guarantee adequate release and efficient acaricidal action. In this context, the incorporation of fipronil into polymeric nanoparticles offers solutions to the compound’s aqueous solubility problems, functioning as carrier vehicles that ensure the protection and optimal delivery of the active ingredient [30,32,40]. However, despite the potential advantages of these nanometric platforms for tick control, their efficacy depends on the proper design and control of critical formulation parameters that guarantee their effectiveness [41]. In this study, fipronil-loaded nanoparticles were prepared using the nanoprecipitation technique with the Eudragit^®^ E PO copolymer, resulting in an average size of 143.43 ± 1.88 nm (Table 1). To date, this polymer had not been used for the formulation of nanoparticles with ixodicide compounds. However, Khachane et al. [42] reported Eudragit^®^ E PO nanoparticles loaded with meloxicam with a size of 95.4 ± 3.2 nm, which demonstrates the versatility of the polymer as a carrier system for active ingredients. On the other hand, previous studies that developed poly(ε-caprolactone) nanoparticles loaded with *Cinnamomum verum* essential oil reported a size of 189.5 nm against *R. microplus* [43], while nanoparticles of the same polymer loaded with thymol reached sizes of 195.7 nm and were evaluated against *R*. *sanguineus* [44]. Although the active ingredients and polymers used differ from those used in this study, the sizes obtained in these formulations are comparable to those presented here. In contrast, Berni et al. [45] developed poly(ε-caprolactone) and chitosan nanoparticles loaded with amitraz and fluazuron, reaching larger sizes of 275 ± 30 nm and 295 ± 35 nm, respectively. These findings highlight the variability in the sizes of nanoparticles evaluated against ticks. However, particle size is a critical parameter, as it directly influences interaction through physical contact, ingestion, or inhalation, as described by García-Ponce et al. [25]. This parameter can also play a decisive role in the release and efficacy of ixodicide compounds, as it promotes interaction with the integument and even enables penetration through the layers of the tick cuticle [25]. On the other hand, PDI is an indicator of the degree of size dispersion of polymeric nanoparticles. This parameter varies between 0 and 1, with values close to 1 reflecting a wide and heterogeneous distribution of sizes, while those close to 0 correspond to monodisperse systems, characterized by minimal variation in particle diameter [46]. In the present study, PDI values of 0.162 ± 0.011 were obtained, which indicates a possible uniformity in the nanoparticles developed. This homogeneity is of great importance, as it could favor interactions between the nanoparticles and the integument of ticks. Another parameter evaluated was Pζ, which is defined as the amount of surface charge present in the nanoparticles and is a determining factor for their colloidal stability [47]. Values greater than +30 mV or less than −30 mV indicate strong electrostatic repulsion between particles, which reduces the possibility of aggregation and improves the stability of the formulations [48,49]. In this study, the fipronil nanoparticles presented a Pζ of 21.16 ± 0.52 mV, a value lower than the limits considered optimal; however, the NP_F nanoformulation remained stable in terms of size, PDI, and Pζ values for 120 days, which suggests adequate resistance to aggregation and good formulation stability (Figure 2). Other important parameters of nanoformulations that must be considered because they allow the active ingredient to be dosed are %EE and %E. In this study, the %EE obtained for the NP_F formulation was 66.28 ± 3.5% (Table 1). This parameter represents the proportion of the active compound effectively incorporated into the nanoparticles relative to the total amount initially used during the formulation process. In a previous study where nanoparticles were prepared with the Eudragit^®^ E PO polymer loaded with meloxicam, an %EE of 89.9 ± 2.1% was obtained [42]. Similarly, the encapsulation of the synthetic acaricides amitraz and fluazuron in poly(ε-caprolactone) and chitosan nanoparticles reported %EE of 77 ± 1% and 89 ± 1%, respectively [45]. These differences could be attributed to the chemical characteristics of the polymers and the encapsulated active ingredients, given that the incorporation of the compound into the nanoparticles depends mainly on the interactions or affinity between the functional groups of the polymer and the active ingredient. For its part, the %E of the NP_F formulation was of 7.36 ± 0.3. Although this value is relatively low, this may be due to the physicochemical characteristics of fipronil, since its hydrophobic nature makes it difficult to incorporate into the nanoformulation matrix.

The FT-IR spectra obtained are shown in Figure 3, providing fundamental information on the possible interactions between fipronil and the components of the nanoformulation. In the spectrum corresponding to pure fipronil, characteristic bands of its structure were identified, which coincide with those previously reported by Kartheek & David [50] and Gajendiran & Abraham [51]. On the other hand, the Eudragit^®^ E PO polymer spectrum showed signals representative of its polymeric unit, consistent with what was described by Jeganathan & Prakya [52]. Regarding the spectra of the nanoparticles without fipronil and the fipronil-loaded nanoparticles, a very similar profile to that of the Eudragit^®^ E PO polymer was observed, with no appearance of new bands or significant shifts. This behavior suggests that no new chemical bonds were formed between fipronil and the polymer, and that the incorporation of the active ingredient into the nanoformulation is based primarily on physical interactions.

On the other hand, resistance to ixodicide principles is a highly relevant issue for livestock production worldwide. In Mexico, populations of *R. microplus* resistant to fipronil [17] have been documented, as well as populations of *Amblyomma mixtum* that are also resistant to this compound [53]. This resistance may originate both from failures in management and application practices (operational factor) and from limitations associated with commercial formulations of fipronil in its pour-on form. Nevertheless, the tick population used in the present study proved susceptible to fipronil, reaching 100% mortality against the DD used. The acaricidal activity of free fipronil against the *R. microplus* population was evidenced by the obtained LC_50_ and LC_90_ values, which were 0.582 µg/mL and 2.503 µg/mL, respectively. These results were compared with those reported by Castro-Janer et al. [36], who determined an LC_50_ of 0.75 µg/mL and an LC_99_ of 2.49 µg/mL for the susceptible Mozo strain, using the LIT. In this context, the LC_50_ obtained in the present study was lower than that reported for the Mozo strain, suggesting that the evaluated population maintains a high susceptibility to fipronil. The fact that the LC_90_ and LC_99_ values are similar between both populations reinforces this conclusion, indicating that the efficacy of the compound is conserved against the evaluated strain. Similarly, the NP_F formulation presented LC_50_ and LC_90_ values of 0.427 µg/mL and 2.092 µg/mL, respectively. These results reflect a decrease compared to free fipronil; however, the statistically significant difference was observed only in the LC_50_. The results obtained suggest that the nanoformulation of fipronil increases its ixodicidal activity, particularly at low concentrations, which translates into greater initial potency against *R. microplus*. This effect could be related to the intrinsic properties of polymeric delivery systems, including their small size and surface nature, which would favor closer interaction with the tick’s integument, expanding the contact surface and facilitating the internalization of the compound, as well as overcoming biological barriers. Consequently, the controlled and sustained release of the active ingredient inside the parasite could prolong its action and contribute to improving the efficacy of the treatment [23,24,25,40,45,54,55]. However, the similarity observed in LC_90_ values suggests that, at higher concentrations, the behavior of free and encapsulated fipronil tends to be comparable. Taken together, these findings support the potential of nanoformulation as a strategy to optimize the use of fipronil by reducing the dose required to obtain significant biological effects, decreasing environmental impact, and mitigating the risk of resistance development in tick populations. Additionally, this formulation offers the advantage of being able to be applied by spraying, which represents an innovative alternative to the limitations of commercial pour-on presentations.

Currently, the development of nanoixodicides or treatments based on nano-carriers of active ingredients with ixodicidal activity is an emerging and innovative area, although still largely unexplored. However, some studies have shown relevant advances. For example, the incorporation of amitraz and fluazuron in polymeric nanoparticles of chitosan and poly-ε-caprolactone showed in in vivo assays against *R. microplus* that the nanoformulation with amitraz not only provided effective protection for 21 days but also had a higher acaricidal potency than conventional commercial products. In addition, both formulations reduced the toxicity of synthetic ixodicides in Balb/c 3T3 cell cultures [45]. Likewise, the larvicidal activity of zein nanoparticles loaded with cypermethrin, chlorpyrifos, and plant-derived compounds (citral, menthol, and limonene) was evaluated, with mortalities exceeding 80% at concentrations of 0.029 mg/mL, as well as efficacy between 40.5 and 60.1% in adult females ingested at the highest concentration tested (0.466 mg/mL). The larvicidal activity of zein nanoparticles loaded with cypermethrin, chlorpyrifos, and plant-derived compounds (citral, menthol, and limonene) was also evaluated, with mortalities greater than 80% observed at concentrations of 0.029 mg/mL and above, as well as efficacies between 40.5 and 60.1% in adult females ingested at the highest concentration tested (0.466 mg/mL) [55]. More recently, it was reported that chitosan nanoparticles loaded with *Eucalyptus globulus* essential oil achieved up to 90% repellency [56]. Taken together, these studies highlight the potential of nanoparticulate systems as an innovative alternative for the development of ixodicidal and repellent treatments. Their ability to deliver diverse active ingredients expands the possibilities for designing more versatile and effective formulations for tick control, while potentially reducing the selective pressure associated with the use of synthetic ixodicides. Nevertheless, their implementation in integrated management programs requires overcoming critical limitations, such as ensuring formulation stability under variable environmental conditions, demonstrating the economic feasibility of large-scale production, and establishing robust in vivo safety profiles that minimize risks to domestic animals, wildlife, and humans. In this context, nanoixodicides represent a highly promising strategy, yet their consolidation demands a multidisciplinary approach that integrates technological innovation, toxicological assessments, and sustainable management practices to ensure long-term efficacy and safety.

## 5. Conclusions

This study demonstrated the feasibility of developing a fipronil-based nanoixodicide encapsulated in Eudragit^®^ E PO nanoparticles (NP_F), which remained stable under ambient conditions for 120 days. The formulation exhibited significantly lower LC_50_ values compared to free fipronil, indicating enhanced efficacy at lower concentrations. In contrast, the similar LC_90_ values suggest that the benefits of nanoencapsulation are mainly evident at reduced dose ranges. Overall, these findings highlight the potential of polymeric nanoparticles to optimize fipronil use, while the possibility of applying NP_F via spraying represents an innovative alternative to conventional commercial pour-on formulations.

## Figures and Tables

**Table 1:** Characterization of fipronil-loaded polymeric nanoparticles and nanoparticles without fipronil (mean ± SD, *n* = 3).

Formulation	Size (nm)	PDI ^a^	ζ Potential (mV)	%E ^b^	%EE ^c^
NP_F ^d^	143.43 ± 1.88	0.162 ± 0.01	21.16 ± 0.54	7.36 ± 0.30	66.28 ± 3.5
NP_B ^e^	119.41 ± 2.05	0.166 ± 0.01	21.81 ± 1.27	-	-

^a^ PDI: Polydispersity index, ^b^ %E: Encapsulation percentage, ^c^ %EE: Encapsulation efficiency percentage, ^d^ NP_F: Fipronil-loaded Eudragit^®^ E PO nanoparticles, ^e^ NP_B: Eudragit^®^ E PO nanoparticles (Without fipronil).

**Table 2:** Resistance/susceptibility status to fipronil of the *R. microplus* tick population.

Treatment	Discriminating Dose	Mortality (%)	Diagnosis
Fipronil	0.05% (p/v)	100 ± 0.00	Susceptible
Control *	--	000 ± 0.00	--

* Control: 10% methanol (*v*/*v*).

**Table 3:** Lethal concentrations at 50% and 90% of the treatments against *Rhipicephalus microplus* larvae.

Treatment	LC_50_ (μg/mL)	CI 95%	LC_90_ (μg/mL)	CI 95%
Fipronil	0.582	0.544–0.622	2.503	2.284–2.767
NP_F	0.427	0.392–0.465	2.092	1.850–2.400

## Data Availability

The original contributions presented in this study are included in the article. Further inquiries can be directed to the corresponding authors.

## Abbreviations

The following abbreviations are used in this manuscript:

NP_F	Nanoparticles loaded with fipronil
NP_B	Nanoparticles without fipronil
PDI	Polydispersity index
Pζ	Z potential
FT-IR	Fourier Transform Infrared Spectroscopy
% E	Encapsulation Percentage
% EE	Encapsulation Efficiency Percentage
DD	Discriminating Dose
LIT	Larval Immersion Test
LC_50_	Lethal Concentration at 50%
LC_90_	Lethal concentration at 90%
LogP	Octanol-water coefficient
